# Occurrence, Bioaccumulation and Dietary Exposure Assessment of Legacy and Emerging Per- and Polyfluoroalkyl Substances (PFAS) in Freshwater Fish from Zhejiang Markets: Implications for Human Health Risks

**DOI:** 10.3390/toxics14060524

**Published:** 2026-06-17

**Authors:** Hexiang Zhang, Yibin Zheng, Xiaojuan Qi, Lili Chen, Jiang Chen, Jikai Wang, Yue He, Bing Zhu

**Affiliations:** 1 Department of Nutrition and Food Safety, Zhejiang Provincial Center for Disease Control and Prevention, Hangzhou 310035, China; hxzhang@cdc.zj.cn (H.Z.); ybzheng@cdc.zj.cn (Y.Z.); xjqi@cdc.zj.cn (X.Q.); llchen@cdc.zj.cn (L.C.); jchen@cdc.zj.cn (J.C.); jkwang@cdc.zj.cn (J.W.); yhe@cdc.zj.cn (Y.H.); 2 NHC Specialty Laboratory of Food Safety Risk Assessment and Standard Development, Hangzhou 310035, China

**Keywords:** per- and polyfluoroalkyl substances, freshwater fish, exposure levels, distribution, bioaccumulation

## Abstract

This article assesses per- and polyfluoroalkyl substances (PFAS) exposure levels, distribution, bioaccumulation characteristics, and health risks in freshwater fish from Zhejiang Province, China. A total of 240 market-sourced fish samples (covering carnivorous, omnivorous, and herbivorous species) collected between 2022 and 2024 were analyzed for 24 PFAS congeners, including four emerging alternatives. The results revealed widespread PFAS contamination, with a detection frequency of 77.5% and median total PFAS (Σ_24_PFAS) concentration of 2.95 ng/g. Hexafluoropropylene oxide dimer acid (HFPO-DA), perfluorooctane sulfonate (PFOS), and perfluoroundecanoic acid (PFUnDA) were the most frequently detected congeners. PFOS exhibited the highest median concentrations in carnivorous and omnivorous fish, whereas PFUnDA was the dominant contaminant in filter-feeding and herbivorous fish. No significant difference in Σ_24_PFAS concentrations was observed among fish with different feeding habits, but highly significant spatial variation was found. Long-chain PFAS concentrations were significantly higher than those of short-chain PFAS and emerging alternatives, with their proportion decreasing at lower trophic levels. The estimated daily intake of Σ_24_PFAS and Σ_4_PFAS for freshwater fish consumers averaged 0.14 and 0.042 ng/kg bw/day, respectively, with approximately 0.16% of the assessed individuals exceeding the recommended threshold. This study provides critical data to support food safety management and risk mitigation strategies.

## 1. Introduction

Per- and polyfluoroalkyl substances (PFAS), a major class of chemical products, have been widely used in a variety of consumer products and industrial processes (e.g., nonstick food packaging, pesticides, lubricants, coating agents, surfactants and fire-fighting foams) [1]. PFAS exhibit properties akin to those of persistent organic pollutants, such as longevity, the ability to bioaccumulate in living organisms, toxicity, and widespread distribution throughout the environment [2]. Due to persistence, bioaccumulation potential, and associated health risks, PFAS use has become a global public health concern. In response, regulatory frameworks have been established worldwide. The European Food Safety Authority (EFSA) set a group tolerable weekly intake (TWI) of 4.4 ng/kg body weight for the sum of perfluorooctanoic acid (PFOA), perfluorononanoic acid (PFNA), perfluorohexanesulfonic acid (PFHxS), and perfluorooctanesulfonic acid (PFOS) [2]. Consequently, European Commission Regulation (EC) 2023/915 set a maximum level of 45 μg/kg wet weight for the sum of these four PFAS in certain freshwater fish products excluding those intended for infants and young children [3]. China included PFOS, PFOA, and their related compounds in the List of Key New Pollutants for Control (2023), prohibiting their production and use, with limited exemption [4].

As traditional PFAS like PFOA and PFOS are increasingly restricted, novel fluorinated alternatives have been introduced. These include hexafluoropropylene oxide dimer acid (HFPO-DA, GenX), 4,8-dioxa-3H-perfluorononanoate (ADONA), and chlorinated polyfluoroalkyl ether sulfonates (e.g., 6:2 Cl-PFESA(F-53B)). These substitutes also pose potential risks to ecosystems and human health [5,6,7,8,9]. Studies have detected emerging PFAS (e.g., F-53B, GenX, ADONA) in environmental and biological samples [5,10]. A recent comprehensive review focusing on China reported that PFOA and PFOS remain the dominant PFAS in human tissues and the environment, with contamination levels generally higher in developed regions; although legacy PFAS show a declining trend, emerging alternatives warrant further investigation [11]. Recent research has increasingly focused on the occurrence of PFAS in various environmental and biological matrices. Comprehensive reviews have systematically documented the levels of PFAS in foodstuffs, highlighting significant concerns regarding dietary exposure. In particular, the consumption of shellfish and freshwater fish has emerged as a primary exposure route for humans, with contaminant levels frequently exceeding established safety thresholds and posing associated health risks [12]. While such studies have established a foundational understanding of PFAS contamination in the general food supply, critical knowledge gaps persist. However, compared to environmental matrices such as water and sediments, research on the bioaccumulation of emerging PFAS (particularly ADONA) in aquatic products—across different species—remains scarce, with limited data on their biomagnification factors. Further investigation is needed to understand their bioaccumulation patterns and health risks. Such work is essential to support evidence-based food safety policies and regulatory standards for these emerging contaminants.

Fish and seafood were found to have relatively high concentrations of PFAS, with major sources of PFOS and PFOA intake related to fish consumption. Previous studies have shown that fish contamination with PFAS is a major health challenge in many areas, such as Greece [13], Finland [14], Vietnam [15], and Netherlands [16]. Numerous studies have revealed that PFAS contamination levels and detection rates in fish highly depend on the capture location and fish species [17,18]. Based on their feeding habits, the fish species were classified into four trophic groups. Filter-feeding fish (e.g., silver carp and bighead carp) filter plankton from the water, occupying lower trophic levels. Herbivorous fish (e.g., bream, grass carp, and Wuchang bream) consume aquatic plants and algae. Omnivorous fish (e.g., yellow catfish, crucian carp) feed on both plant and animal matter. Carnivorous fish (e.g., mandarinfish, black carp) prey on other fish and aquatic invertebrates, occupying the highest trophic levels with the greatest potential for PFAS biomagnification.

Zhejiang Province is located in the southeast coast of China, south of the Yangtze River delta, east of the East China Sea, where freshwater fish is an important part of the daily diet of the local people. Nevertheless, there remains a notable scarcity of comprehensive studies focusing on commercially available freshwater fish—a major dietary source—that simultaneously investigate both legacy and emerging PFAS. Existing risk assessments of PFAS exposure via freshwater fish consumption in other regions have often relied on averaged rather than individualized consumption and body weight data [14,15], and have typically included only a limited spectrum of PFAS compounds in small sample sets [18,19]. These limitations introduce considerable uncertainty and underscore the fact that the true extent of human exposure and associated health risks for the local population remains poorly understood.

The objectives of this study were therefore: (1) to determine the concentrations and compositional profiles of 24 legacy and emerging PFAS in the edible tissues of commercially important freshwater fish species from local markets in Zhejiang Province; (2) to investigate the bioaccumulation patterns of these PFAS across species with different feeding habits (herbivorous, carnivorous, filter-feeding and omnivorous); (3) to estimate the dietary exposure levels of the general population (including adults and children) through freshwater fish consumption; and (4) to assess the potential health risks based on a comparison with the tolerable daily intake of 0.63 ng/kg bw per day for Σ_4_PFAS (PFOA, PFNA, PFHxS, and PFOS) [2].

## 2. Materials and Methods

### 2.1. Sample Collection and Preparation

**Figure 1:**
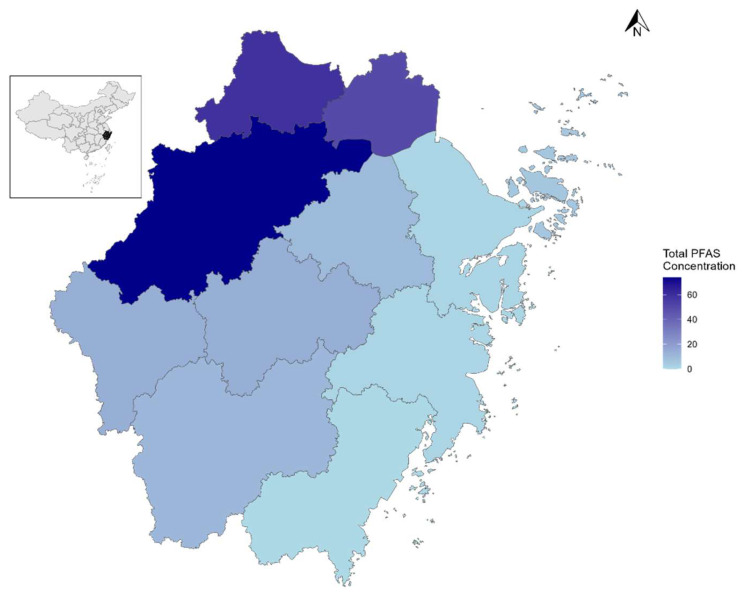
Map of the sampling locations for freshwater fish in Zhejiang.

During the period of 2022–2024, 240 freshwater fish samples were collected in Zhejiang Province (Figure 1). The freshwater fish included carnivorous (*n* = 82), omnivorous (*n* = 98), herbivorous (*n* = 37) and filter-feeding (*n* = 23) species. The carnivorous freshwater fish comprised Chinese perch, river perch, river eel, northern snakehead, Asian swamp eel, sea bass, black carp, marble goby, sturgeon, and black seabream. The omnivorous freshwater fish species included Ussuri sharpbelly, yellow catfish, white bream, crucian carp, bass, tilapia, Chinese hooksnout carp, oriental weatherfish, jaguar cichlid (freshwater “grouper”), Chinese barb, and flathead grey mullet. The herbivorous freshwater fish species included white bream, grass carp, and Wuchang bream. The filter-feeding species included bighead carp and silver carp. These collected freshwater fish species were most consumed by locals. Trained investigators were instructed to purchase duplicate samples (approximately 1.0 kg) of each of the freshwater fish from farmers’ markets, retail shops, supermarkets, aquaculture farms and online stores.

After collection, non-edible parts of the freshwater fish, including viscera and scales, were removed at the point of sale and immediately transported to the laboratory under controlled temperature conditions (4 °C) in an insulated container. The samples were rinsed three times with ultrapure water. The edible portions of the fish samples were homogenized using a blender and stored in a freezer at or below −18 °C pending analysis.

### 2.2. Instrumental Analysis and Quality Assurance

The target analytes in this study comprised 24 per- and polyfluoroalkyl substances (PFAS), including 13 perfluorocarboxylic acids (PFCAs, C4-C16 and C18), 7 perfluorosulfonic acids (PFSAs, C4-C10), and 4 emerging alternatives: 6:2 Cl-PFESA (F-53B), 8:2 Cl-PFESA, HFPO-DA (Gen-X), and ADONA. Abbreviations are provided in Table S1.

Sample collection and methods applied covering sample preparation, extraction, clean-up, and instrumental analysis have been described elsewhere. Specifically, the target list was expanded from 21 to 24 PFAS by incorporating 6:2 Cl-PFESA (6/2 F-53), 8:2 Cl-PFESA (8/2 F-53), and HFPO-DA (Gen-X). All other aspects of the methodology, including the quality assurance/quality control protocols (e.g., use of isotope-labeled internal standards, recovery tests, and blank monitoring), remained consistent with the previously published approach [20]. In summary, the edible portions of aquatic products were cut into small pieces and homogenized using a tissue homogenizer. A 2.0 g portion of the aquatic sample was placed into a 50 mL polypropylene tube. The isotope internal standard working solution (2 ng isotopically labeled PFAS; Wellington Laboratories, Guelph, ON, Canada) was added, followed by 2.0 mL of ultrapure water and 8.0 mL of acetonitrile. The mixture was thoroughly mixed and sonicated for 30 min. Then, the entire supernatant was collected, and the extract was purified using an HLB-P/HMR-Lipid solid-phase extraction column (Anavo, Beijing, China). All eluate was collected, and 1.5 g of sodium chloride was added, mixed thoroughly, and shaken to achieve phase separation. The mixture was centrifuged at 10,000 rpm for 10 min. Then, 4.0 mL of the upper acetonitrile layer was transferred, evaporated to dryness using nitrogen, and diluted to 0.2 mL with methanol. Quantification was performed by isotope dilution using an ultra-performance liquid chromatography/tandem mass spectrometry instrument (Xevo TQ-S; Waters, Milford, MA, USA).

In the present study, the limit of detections (LODs) of the three additional PFAS were 0.01 ng/g for 6:2 Cl-PFESA, 8:2 Cl-PFESA, and HFPO-DA. The left-censored data (concentration results below LOD) were treated using the substitution method as recommended in the “Principles and Methods for the Risk Assessment of Chemicals in Food” [21], that is, concentrations of all ND were set to 1/2 LOD.

### 2.3. Food Consumption Data

Food consumption data for the Zhejiang population were obtained from the Zhejiang Comprehensive Food Consumption Database [20]. In summary, each participant underwent three non-consecutive 24 h recall surveys to collect information on aquatic product consumption within the 24 h prior to the survey date, and review the frequency of freshwater fish consumption within the past year. Children under 12 years old could be assisted by parents or primary caregivers. Body weights of individuals were measured simultaneously. All participants signed an informed consent form and their personal information was kept confidential.

After excluding individuals who did not consume freshwater fish or had missing data (such as age and weight), 6763 freshwater fish consumers were selected for inclusion in this study. The consumption and body weight data were categorized into five age groups: young children (3–6 years), older children (7–13 years), adolescents (14–17 years), adults (18–60 years), and older adults (≥60 years).

### 2.4. Risk Assessment

To evaluate the health risks of individuals in Zhejiang Province via freshwater fish consumption, the estimated daily intake of the target PFAS (EDI: ng/kg bw/day) was calculated using Formulas (1) [22] and (2).

(1) $${\mathrm{EDI}}_{i\;}=\;\frac{C_{i}\;\times \;{\mathrm{IR}}_{i}\;\times \;{\mathrm{EF}}_{i}\;\times \;\mathrm{ED}}{\mathrm{BW}\;\times \;\mathrm{AT}}$$

(2) $$\mathrm{EDI}=\sum {\mathrm{EDI}}_{i}$$

(3) $$\mathrm{HQ}=\frac{\mathrm{EDI}\;\times \;7}{\mathrm{TWI}}$$

In Formula (1) EDI_i_ represent the estimated daily intake of PFAS for an individual through freshwater fish species i, where:

C_i_ = The P50 of concentration of the target PFAS in freshwater fish species i.
IR_i_ = Individual’s daily consumption of freshwater fish species I (g/day).
EF_i_ = Exposure frequency of fish species i (days/year).
AT = Averaging time for non-carcinogenic effects (365 days/year × ED).
BW = The weight of the individual’s actual body (kg).

In Formula (2), EDI represents the individual estimated daily intake by all of the freshwater fish species.

In Formula (3), the tolerable weekly intake (TWI) (ng/kg·bw·d) is the recommended reference dose, and HQ is the hazard quotient. TWI was set to 4.4 ng/kg body weight per week (equivalent to 0.63 ng/kg body weight per day), specifically, for the sum of PFOS, PFOA, PFNA, and PFHxS [2]. A HQ ≥ 1 was defined to indicate an unacceptable exposure risk resistance in the population.

### 2.5. Statistical Analysis

The Shapiro−Wilk test was conducted to test the normality of the PFAS data. The Wilcoxon test was performed to compare the differences between the two groups. A Kruskal–Wallis test was conducted to compare the differences among the three groups. If a significant difference was identified, pairwise comparisons were then performed using Dunn’s test to determine which pairs of origins differed most significantly. *p*-values were adjusted using the Bonferroni method for multiple comparisons. Spearman correlation was used for correlation analysis. Data were statistically analyzed and the graphics were drawn using R Statistical Software (version 4.4.2; R Foundation for Statistical Computing, Vienna, Austria). The significance level was set at *p*-value < 0.05.

## 3. Results

### 3.1. Overall Pollution Levels and Composition Profiles of PFAS

**Figure 2:**
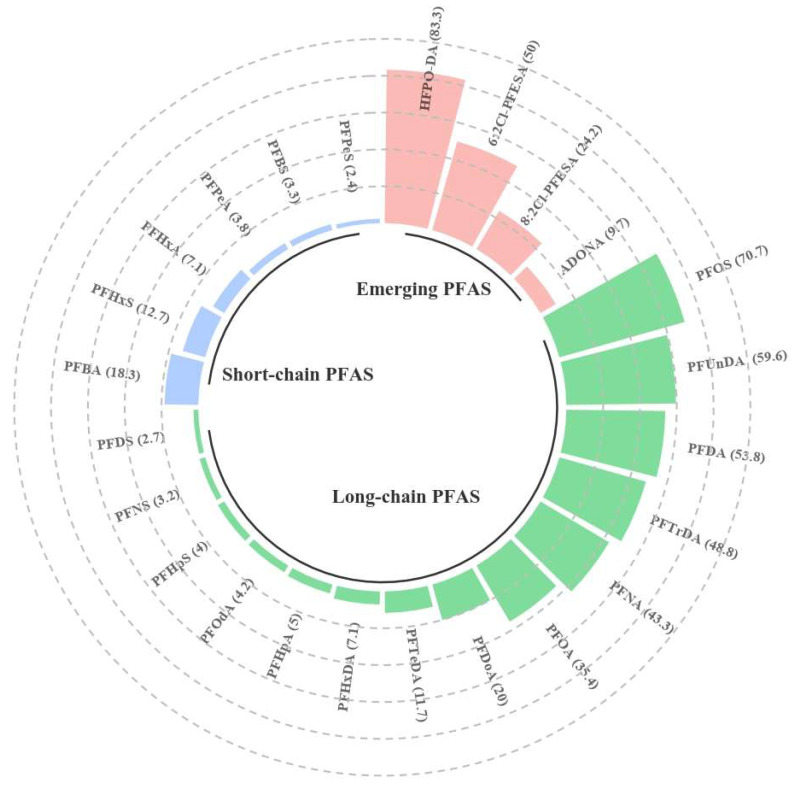
The detection rates (%) of Σ_24_PFAS in freshwater fish. Red represents emerging PFAS, green represents long-chain PFAS, and blue represents short-chain PFAS.

The occurrence of 24 target PFAS was investigated in the edible tissues of freshwater fish. The overall detection frequency of Σ_24_PFAS was 77.5% (186 out of 240 samples). As illustrated in Figure 2, marked differences in detection rates were observed among individual congeners, ranging from 2.38% to 83.33%. The concentrations of Σ_24_PFAS ranged from not detected (ND) to 71.6 ng/g wet weight, with a mean of 5.1 ng/g and a median (P50) of 2.95 ng/g.

#### 3.1.1. Profiles of Legacy and Emerging PFAS (Figure 2)

Among the legacy PFAS, PFOS, PFUnDA, and PFDA were the most frequently detected, with detection rates of 70.7%, 59.6%, and 53.8%, respectively. Analysis of four emerging alternatives—HFPO-DA (Gen-X), ADONA, 6:2 Cl-PFESA (F-53B), and 8:2 Cl-PFESA—revealed distinct detection patterns. HFPO-DA showed the highest detection frequency (83.3%) and a median concentration of 0.12 ng/g (range: ND–0.22 ng/g). This was followed by 6:2 Cl-PFESA, detected in 50.00% of samples but at a lower median concentration of 0.01 ng/g (range: ND–4.92 ng/g). In contrast, ADONA and 8:2 Cl-PFESA exhibited considerably lower detection rates (9.68% and 24.9%, respectively), both with median concentrations of 0.01 ng/g. The detailed information of these PFAS were presented in Table S2. This heterogeneous pattern indicates that HFPO-DA and 6:2 Cl-PFESA are currently the predominant emerging PFAS in the studied region.

#### 3.1.2. Dominant Congeners: Mass Burden Versus Typical Concentration

Notably, the ranking of dominant PFAS congeners differed depending on the metric applied. PFBA, PFUnDA, and PFOS were the three largest contributors to the total Σ_24_PFAS mass burden, accounting for 28.4%, 15.7%, and 12.5%, respectively. However, when ranked by the median concentration across all samples—a metric reflecting typical exposure levels—PFOS, PFUnDA, PFDA, PFTrDA, and PFNA emerged as the top five. This discrepancy highlights that compounds contributing most to the total pollution mass (e.g., PFBA) are not necessarily those found at consistently high concentrations in every sample, potentially due to sporadic, high-concentration occurrences in specific specimens or significant regional heterogeneity.

### 3.2. Σ_24_PFAS Contamination Profiles Across Fish with Different Feeding Habits

**Figure 3:**
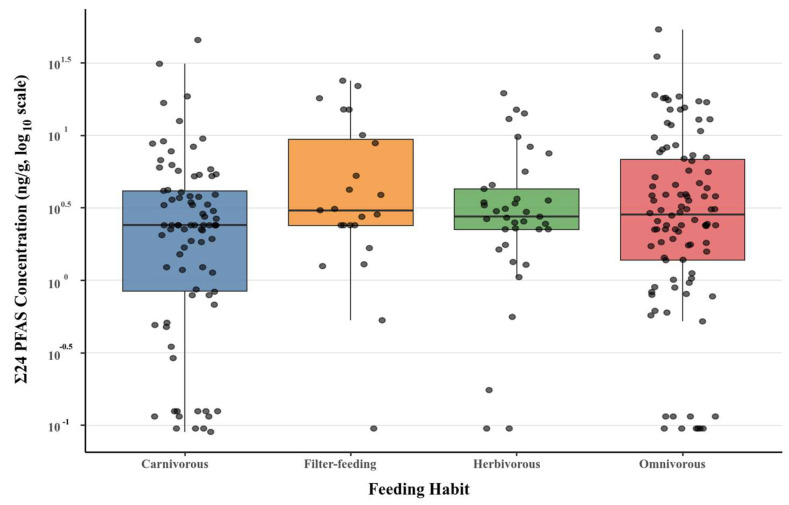
Σ_24_ PFAS concentrations among fish with different feeding habits. Boxplots indicate median, interquartile range (IQR); jittered dots show individual measurements. Colors correspond to feeding habit groups.

Based on their feeding habits, fish were categorized into four groups: carnivorous, omnivorous, filter-feeding and herbivorous. Overall, the differences in Σ_24_PFAS concentrations among the four groups were not statistically significant (*p* = 0.192) (Figure 3). In contrast, a highly significant difference in Σ_24_ PFAS concentrations was found among fish from different geographical origins (*p* = 1.80 × 10^−6^). To further identify the source of variation, pairwise comparisons were conducted using Dunn’s post hoc test. The results revealed statistically significant differences between several location pairs, with the most pronounced difference observed between fish from Lishui and Jiaxing (*p* = 0.00032). Significant differences were also detected between Lishui and Huzhou (*p* = 0.00153), as well as between Lishui and fish of unspecified origin (*p* = 0.00032).

### 3.3. Differences in the Composition of Legacy and Emerging PFAS in Freshwater Fish

Differences in the composition of legacy and emerging per- and polyfluoroalkyl substances (PFAS) were analyzed in freshwater fish. Legacy PFAS were further classified into short-chain (≤6 carbons) and long-chain (≥7 carbons) categories. The result indicated significant concentration differences between all chain-length groups (all *p* < 0.001), with the most pronounced difference observed between long-chain and emerging PFAS. Long-chain PFAs exhibited the highest median concentration (0.0484 ng/g), which was significantly greater than those of short-chain (0.0100 ng/g) and emerging PFAS (0.0050 ng/g). Specifically, the median concentration of long-chain PFAS was 4.8 and 9.7 times higher than that of short-chain and emerging PFAS, respectively.

In the relative abundance of PFAS in freshwater fish with different feeding habits, the proportion of long-chain PFAS decreased progressively from carnivorous (75.0%) to omnivorous (59.2%), filter-feeding (56.6%), and herbivorous (50.5%) fish. Conversely, short-chain PFAS increased along the same gradient (21.8% → 36.6% → 43.2% → 43.1%). Long-chain legacy PFAS consistently dominated across all groups (median: 77.5%, 59.3%, 56.6%, and 50.5%, respectively), while emerging PFAS were commonly detected but accounted for only a low proportion (median range: 0.269–4.35%).

### 3.4. Species-Dependent Accumulation of Major PFAS Monomers

**Figure 4:**
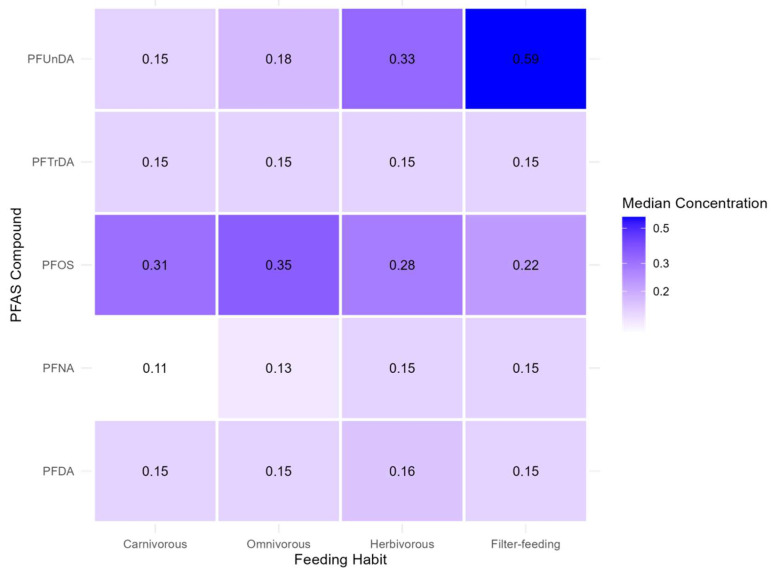
Heatmap of key PFAS monomer concentrations in different species.

The heatmap, based on median concentrations of PFAS monomers, identified the five most abundant compounds across fish of different feeding habits: PFOS, PFUnDA, PFDA, PFTrDA, and PFNA (Figure 4). As indicated in the heatmap, PFOS exhibited the highest median concentrations in carnivorous (0.31 ng/g) and omnivorous (0.35 ng/g) fish. This pattern suggests a pronounced trophic magnification potential for PFOS in higher trophic-level species. In contrast, PFUnDA emerged as the dominant contaminant in filter-feeding and herbivorous fish, with a median concentration of 0.59 ng/g and 0.33 ng/g, respectively, indicating a distinct bioaccumulation profile compared to PFOS. The remaining high concentration PFAS monomers—PFDA, PFTrDA, and PFNA—showed less pronounced variation across feeding guilds, with median concentrations ranging between 0.11 and 0.15 ng/g across all groups. These three monomers exhibited relatively similar contamination patterns across freshwater fish of various feeding habits.

### 3.5. Chronic Dietary Exposure and Risk Assessment

Among all detected substances, the median exposure levels of traditional PFAS were generally higher than those of emerging PFAS (Table S3). Specifically, long-chain traditional PFAS, including PFUnDA (median: 0.0120 ng/kg bw/day), PFOS (0.00904 ng/kg bw/day), PFDA (0.00885 ng/kg bw/day), and PFNA (0.00768 ng/kg bw/day), dominated the total traditional PFAS exposure. PFOA showed a median level of 0.00434 ng/kg bw/day, falling into an intermediate range. Short-chain compounds such as PFBA (0.00256 ng/kg bw/day), PFPeA (0.000722 ng/kg bw/day), and PFHxA (0.000370 ng/kg bw/day) exhibited relatively lower levels. Among emerging PFAS, HFPO-DA had the highest median concentration (0.00499 ng/kg bw/day), yet it remained below the levels of the major traditional PFAS (PFUnDA, PFOS, PFDA, and PFNA). The medians of 6:2Cl-PFESA and 8:2Cl-PFESA were 0.00108 and 0.000294 ng/kg bw/day, respectively, with ADONA showing the lowest median (0.000280 ng/kg bw/day). None of the emerging PFAS exceeded the top four traditional PFAS in terms of median concentration.

The estimated total daily intake (EDI) values for Σ_24_PFAS via freshwater fish consumption ranged from mean 0.14 to maximal 12.2 ng/kg bw/day and a 95th percentile (P95) of 0.47 ng/kg bw/day (Table S4). For Σ_4_PFAS, the EDI ranged from mean of 0.042 ng/kg bw/day to maximal 2.09 ng/kg bw/day, with a P95 of 0.14 ng/kg bw/day (Table S5). The highest Σ_24_PFAS intake occurred in adults (18–59 years), while the maximum Σ_4_PFAS intake was observed in older adults (≥60 years). Exposure levels of both Σ_24_PFAS (Figure S1) and Σ_4_PFAS (Figure S2) showed no significant differences between young children (3–6 years) and older children (7–13 years), nor between adolescents (14–17 years) and adults. Overall, dietary exposure to PFAS from freshwater fish exhibited a decreasing trend with age. Nearly 0.16% (11 out of 6763) of the study population had estimated exposure levels exceeding the recommended TWI.

## 4. Discussion

This study identified PFAS contamination in freshwater fish from the Zhejiang market, with a total of 24 target compounds analyzed. The total concentration ranged from ND to 71.6 ng/g ww, which was higher than the levels reported for nine PFAS in freshwater fish from northern Vietnam (0.08–8.06 ng/g ww) [15] and higher than the total concentrations of 12 PFAS detected in freshwater fish from Beijing (1.70–14.32 ng/g ww) [23]. However, the levels were lower than those reported for freshwater fish from the Jiulong River in China (25–100 ng/g ww) [24] and for fish from New Jersey, USA (3.8–129.8 ng/g ww) [25]. The mean concentration in this study (5.10 ng/g ww) was lower than the mean concentrations of 11 PFAS in fish filets from Charleston Harbor, USA (6.2–12.7 ng/g ww) [19], and the median concentration (2.95 ng/g ww) was lower than that reported for European lakes (median 14.2 ng/g ww) [26]. Although direct comparisons are limited by differences in the PFAS analyte lists, the overall PFAS contamination levels in freshwater fish from the Zhejiang market can be considered moderate to low on a global scale, and notably lower than those observed in highly polluted regions of Europe and industrialized watersheds in the United States.

Among the long-chain PFAS, PFOS, PFDA, PFUnDA, PFTrDA, and PFNA are the main contributors. The PFAS profiles observed in this study exhibit both similarities and differences compared to those reported in other regions globally. The predominance of PFOS and long-chain perfluoroalkyl carboxylic acids (PFCAs), such as PFUnDA and PFDA, in freshwater fish from Zhejiang aligns with findings from several previous studies. For instance, PFOS dominance has been widely documented in freshwater fish from various locations, including Taihu Lake in China [27] and New Jersey, USA [25]. Similarly, the high contribution of PFUnDA and PFDA observed here is consistent with profiles reported in fish from European lakes [26], Vietnam [15], the Veneto Region, and Italy [28], suggesting a widespread distribution and bioaccumulation potential of these long-chain PFAS in freshwater ecosystems globally. However, the PFAS profile in Zhejiang fish differed substantially from the PFHxA-dominant profile in Lake Chaohu [29], highlighting the influence of localized contamination sources. These discrepancies likely stem from regional differences in industrial activities, wastewater discharge, and hydrological conditions.

In this study, although no significant differences were observed in the total concentrations of the 24 PFAS among fish with different feeding habits, the bioaccumulation concentrations of long-chain PFAS were significantly higher than those of short-chain homologs and novel alternatives, and exhibited a clear decreasing trend with declining trophic level. This finding aligns with the results of a global study, which reported that the biomagnification potential of long-chain PFAS (e.g., PFOS, PFDA, PFUnDA) underscores their potential contribution along the food chain [30]. Furthermore, the study also revealed that the proportion of short-chain PFAS increased significantly as trophic level decreased, accounting for over 40% of total PFAS in herbivorous fish. Biomagnification of short-chain PFAAs were also observed in lower trophic levels of the food web [31]. This suggests that the contribution of short-chain PFAS cannot be overlooked when assessing the health risks associated with low-trophic-level aquatic products. These findings are closely related to food safety concerns. Although long-chain PFAS compounds such as PFOS have garnered widespread attention due to their persistence and significant bioaccumulation capacity through the food chain, short-chain PFAS can also be transferred and accumulated along the food chain, thereby further exacerbating human exposure risks. Their environmental fate and toxicological effects thus warrant equal attention.

Regarding the four emerging PFAS analyzed in this study, notably high detection rates were observed for HFPO-DA (Gen-X) and 6:2 Cl-PFESA (F-53B), despite their low contribution to the total PFAS burden. This pattern—high detection frequency with low concentration—suggests that these alternatives have already entered the aquatic environment and been taken up by fish, but have not yet become dominant contaminants. Notably, the frequent detection of emerging PFAS alternatives, such as F-53B and Gen-X, may reflect a direct environmental consequence of China’s increased production and application of these substitutes following the listing of PFOS under the Stockholm Convention. Their widespread presence—documented across rivers in China and multiple European countries [32,33]—underscores a global distribution pattern. The relatively high detection rate of 6:2 Cl-PFESA (F-53B) is consistent with a previous study in Taihu Lake [27], indicating its increasing prevalence as a substitute for PFOS in Chinese freshwater ecosystems. Despite the small contribution of F-53B and Gen-X at current concentrations, their potential ecological risks are not to be ignored. A global consensus analysis reveals that F-53B exhibits a trophic magnification factor (TMF) of 3.07 in all 72 PFAS, ranking first among them and even surpassing PFOS’s TMF of 3.02 [28]. This finding underscores a critical concern: compounds with low current concentrations but high bioaccumulation potential may pose delayed risks as their environmental release continues. Given the unknown toxicological effects and potential bioaccumulation of these emerging PFAS, continued monitoring is warranted.

The EDI of Σ_4_PFAS via freshwater fish consumption in the present study (mean: 0.042 ng/kg bw/day, 95th percentile: 0.14 ng/kg bw/day) was comparable to values reported for other populations. For instance, the general population EDI in Singapore (mean: 0.0031 ng/kg bw/day, 95th percentile: 0.17 ng/kg bw/day) was within the same order of magnitude as our estimate, with fish and seafood accounting for 89.7% of total PFAS exposure [34]. Likewise, the Σ_4_PFAS intake from aquatic products ranged from 0.54 ng/kg bw/day (Shanghai elderly) to 0.55 ng/kg bw/day (Liaoning adults) [17,35], all of which were comparable to our P95 level (0.14 ng/kg bw/day). The EDI values in the present study were substantially lower than those reported for Vietnamese adults via freshwater fish consumption (0.38–4.76 ng/kg bw/day) [15]. Discrepancies in EDI values across studies can be primarily attributed to differences in PFAS contamination levels in aquatic products, the inclusion of different categories (e.g., freshwater fish versus shellfish/crustaceans/seafood), and the methodological approach to consumption frequency. Notably, the exposure estimates cited above were calculated without incorporating aquatic product consumption frequency data—namely, the number of times aquatic products are consumed per year—owing to the challenges in obtaining reliable consumption data. The absence of this variable may lead to over- or underestimation of actual intake, especially in populations with irregular or seasonal consumption patterns.

## 5. Conclusions

This study evaluated the contamination levels, bioaccumulation potential, and human exposure risks of legacy and emerging PFAS in freshwater fish sold in Zhejiang markets. Based on data collected from 2022 to 2024, PFAS were commonly detected in freshwater fish, with HFPO-DA, PFOS, and PFUnDA being the most frequently detected congeners. Nevertheless, PFOS, PFUnDA, PFDA, PFTrDA, and PFNA were identified as the five congeners of greatest concern. Long-chain PFAS predominated the contamination profile in freshwater fish. Spatial variations in PFAS distribution were observed, and differences in PFAS composition were noted among fish with different feeding habits; however, no significant difference was found in total PFAS concentrations. Dietary exposure assessment indicated a low risk from exposure to four PFAS congeners via freshwater fish consumption. Nevertheless, the potential risk associated with chronic exposure to multiple PFAS warrants consideration. Notably, despite the relatively low levels of PFAS in freshwater fish, their persistence over time, the absence of established reference doses for combined exposure to mixtures of additional PFAS congeners, and the health risks posed by cumulative exposure through multiple food items remain unaddressed. Furthermore, research on the interactive effects between PFAS and other persistent pollutants is limited. Therefore, the data generated in this study can serve as a scientific basis for informing future regulatory policy development.

## Data Availability

The raw data supporting the conclusions of this article will be made available by the authors on request.

## Supplementary Materials

The following supporting information can be downloaded at: https://www.mdpi.com/article/10.3390/toxics14060524/s1, Table S1: Pollutant classification and corresponding LOD and LOQ values; Table S2: Levels of PFAS congeners in freshwater fish from Zhejiang Province; Table S3: The estimated EDI for PFAS congeners; Table S4: The estimated EDI for Σ_24_PFAS dietary exposure; Table S5: The estimated EDI for Σ_4_PFAS dietary exposure. Figure S1: Comparison of Σ_24_PFAS exposure levels (ng/kg bw per day) among different age groups; Figure S2: Comparison of Σ_4_PFAS exposure levels (ng/kg bw per day) among different age groups.

## References

[1] Prevedouros K., Cousins I.T., Buck R.C., Korzeniowski S.H. (2006). Sources, fate and transport of perfluorocarboxylates. Environ. Sci. Technol..

[2] Schrenk D., Bignami M., Bodin L., Chipman J.K., del Mazo J., Grasl-Kraupp B., Hogstrand C., Hoogenboom L., Leblanc J., EFSA Panel on Contaminants in the Food Chain (EFSA CONTAM Panel) (2020). Risk to Human Health Related to the Presence of Perfluoroalkyl Substances in Food. EFSA J..

[3] European Commission (2023). Commission Regulation (EU) 2023/915 of 25 April 2023 on maximum levels for certain contaminants in food and repealing Regulation (EC) No 1881/2006. Off. J. Eur. Union.

[4] Ministry of Ecology and Environment (2023). List of Key Controlled New Pollutants (2023 Edition).

[5] He Y., Lv D., Li C., Liu X., Liu W., Han W. (2022). Human exposure to F-53B in China and the evaluation of its potential toxicity: An overview. Environ. Int..

[6] Robarts D.R., Venneman K.K., Gunewardena S., Apte U. (2022). GenX induces fibroinflammatory gene expression in primary human hepatocytes. Toxicology.

[7] Liang L.X., Liang J., Li Q.Q., Zeeshan M., Zhang Z., Jin N., Lin L.Z., Wu L.Y., Sun M.K., Tan W.H. (2023). Early life exposure to F-53B induces neurobehavioral changes in developing children and disturbs dopamine-dependent synaptic signaling in weaning mice. Environ. Int..

[8] Wu S., Xie J., Zhao H., Sanchez O., Zhao X., Freeman J.L., Yuan C. (2023). Pre-differentiation GenX exposure induced neurotoxicity in human dopaminergic-like neurons. Chemosphere.

[9] Feng L., Lang Y., Feng Y., Tang X., Zhang Q., Xu H., Liu Y. (2024). Maternal F-53B exposure during pregnancy and lactation affects bone growth and development in male offspring. Ecotoxicol. Environ. Saf..

[10] Guo W., Hao W., Xiao W. (2024). Emerging Perfluorinated Chemical GenX: Environmental and Biological Fates and Biological Fates and Risks. Environ. Health.

[11] Li J., Duan W., An Z., Jiang Z., Li L., Guo M., Tan Z., Zeng X., Liu X., Liu Y. (2024). Legacy and alternative per- and polyfluoroalkyl substances spatiotemporal distribution in China: Human exposure, environmental media, and risk assessment. J. Hazard. Mater..

[12] Souza M.C.O., Domingo J.L. (2025). Levels of per- and polyfluoroalkyl substances (PFAS) in foodstuffs: A review of dietary exposure, health risks, and regulatory challenges. Food Res. Int..

[13] Costopoulou D., Vassiliadou I., Leondiadis L. (2022). PFASs intake from fish, eggs and drinking water in Greece in relation to the safety limits for weekly intake proposed in the EFSA scientific opinion of 2020. Chemosphere.

[14] Kumar E., Koponen J., Rantakokko P., Airaksinen R., Ruokojärvi P., Kiviranta H., JVuorinen P., Myllylä T., Keinänen M., Raitaniemi J. (2022). Distribution of perfluoroalkyl acids in fish species from the Baltic Sea and freshwaters in Finland. Chemosphere.

[15] Vi P.T., Ngoc N.T., Quang P.D., Dam N.T., Tue N.M., Tuyen L.H., Viet P.H., Anh D.H. (2022). Perfluoroalkyl substances in freshwater and marine fish from northern Vietnam: Accumulation levels, profiles, and implications for human consumption. Mar. Pollut. Bull..

[16] Zafeiraki E., Gebbink W.A., Hoogenboom R.L.A.P., Kotterman M., Kwadijk C., Dassenakis E., van Leeuwen S.P.J. (2019). Occurrence of perfluoroalkyl substances (PFASs) in a large number of wild and farmed aquatic animals collected in the Netherlands. Chemosphere.

[17] Zhang M., Cai D., Chen X.X., Sun Y.N., Pan J., Ding P., Li T.Z., Hu G.C. (2024). Contamination characteristics and health risk assessment of poly-and perfluoroalkyl substances and alternatives in fish from Liaoning Province. Environ. Chem..

[18] Ali A.M., Sanden M., Higgins C.P., Hale S.E., Alarif W.M., Al-Lihaibi S.S., Ræder E.M., Langberg H.A., Kallenborn R. (2021). Legacy and emerging per- and polyfluorinated alkyl substances (PFASs) in sediment and edible fish from the Eastern Red Sea. Environ. Pollut..

[19] Fair P.A., Wolf B., White N.D., Arnott S.A., Kannan K., Karthikraj R., Vena J.E. (2019). Perfluoroalkyl substances (PFASs) in edible fish species from Charleston Harbor and tributaries, South Carolina, United States: Exposure and risk assessment. Environ. Res..

[20] Zhang H., Zhang H., Zhang R., Zhao D., Zhu B., Qi X., Chen L., Chen J., Wang J., Zheng Y. (2025). Contamination Characteristics of 21 PFAS in Shellfish and Crustaceans of Zhejiang Province and Exposure Risk Assessment for Adult Dietary Consumers. Mar. Drugs.

[21] WHO/IPCS (World Health Organization/International Programme on Chemical Safety) (2009). Environmental Health Criteria 240: Principles and Methods for the Assessment of Chemicals in Food.

[22] U.S. Environmental Protection Agency Regional Screening Levels (RSLs)—User’s Guide. https://www.epa.gov/risk/regional-screening-levels-rsls-users-guide#target.

[23] Liu S.F., Wang T.Y., Xue K.S., Wang P., Sun Y.J. (2017). Occurrence and human health risk of PFASs in fishes from drinking water sources of Beijing. Asian J. Ecotoxicol..

[24] Wang S., Cai Y., Ma L., Lin X., Li Q., Li Y., Wang X. (2020). Perfluoroalkyl substances in water, sediment, and fish from a subtropical river of China: Environmental behaviors and potential risk. Chemosphere.

[25] Goodrow S.M., Ruppel B., Lippincott R.L., Post G.B., Procopio N.A. (2020). Investigation of levels of perfluoroalkyl substances in surface water, sediment and fish tissue in New Jersey, USA. Sci. Total Environ..

[26] Valsecchi S., Babut M., Mazzoni M., Pascariello S., Ferrario C., De Felice B., Bettinetti R., Veyrand B., Marchand P., Polesello S. (2021). Per- and Polyfluoroalkyl Substances (PFAS) in Fish from European Lakes: Current Contamination Status, Sources, and Perspectives for Monitoring. Environ. Toxicol. Chem..

[27] Chen M., Zhu L., Wang Q., Shan G. (2021). Tissue distribution and bioaccumulation of legacy and emerging per-and polyfluoroalkyl substances (PFASs) in edible fishes from Taihu Lake, China. Environ. Pollut..

[28] Gallocchio F., Mancin M., Belluco S., Moressa A., Angeletti R., Lorenzetto M., Arcangeli G., Ferrè N., Ricci A., Russo F. (2022). Investigation of levels of perfluoroalkyl substances in freshwater fishes collected in a contaminated area of Veneto Region, Italy. Environ. Sci. Pollut. Res. Int..

[29] Wu J.Y., Liu W.X., He W., Xu F.L. (2019). Comparisons of tissue distributions and health risks of perfluoroalkyl acids (PFAAs) in two fish species with different trophic levels from Lake Chaohu, China. Ecotoxicol. Environ. Saf..

[30] Ricolfi L., Yang Y., Pottier P., Morrison K., Williams C., Pollo P., Hesselson D., Neely G.G., Taylor M.D., Nakagawa S. (2025). Unravelling the magnitude and drivers of PFAS trophic magnification: A meta-analysis. Nat. Commun..

[31] Chu K., Lu Y., Hua Z., Liu Y., Ma Y., Gu L., Gao C., Yu L., Wang Y. (2022). Perfluoroalkyl acids (PFAAs) in the aquatic food web of a temperate urban lake in East China: Bioaccumulation, biomagnification, and probabilistic human health risk. Environ. Pollut..

[32] Joerss H., Schramm T.R., Sun L., Guo C., Tang J., Ebinghaus R. (2020). Per- and polyfluoroalkyl substances in Chinese and German river water—Point source- and country-specific fingerprints including unknown precursors. Environ. Pollut..

[33] Liu S., Jin B., Arp H.P.H., Chen W., Liu Y., Zhang G. (2022). The Fate and Transport of Chlorinated Polyfluorinated Ether Sulfonates and Other PFAS through Industrial Wastewater Treatment Facilities in China. Environ. Sci. Technol..

[34] Lim I., Shen P., Ang W.M., Chin Y.S., Shi R.R.S., Yu W.Z., Chan S.H. (2025). Occurrence and Dietary Exposure of PFAS in Singapore: Insights from a Total Diet Study. Foods.

[35] Li T., Wang Y., Wu X.H., Chen J., Lin N., Feng N. (2024). Risk Assessment of Ingestion Exposure to Per-and Poly-fluoroalkyl Substances among Elderly People in a Community in Shanghai. Asian J. Ecotoxicol..

