# Supplementary material for: Occurrence, Bioaccumulation and Dietary Exposure Assessment of Legacy and Emerging Per- and Polyfluoroalkyl Substances (PFAS) in Freshwater Fish from Zhejiang Markets: Implications for Human Health Risks
Source: Toxics. 2026 Jun 17;14(6):524. doi: 10.3390/toxics14060524 (PMC13307121; doi:10.3390/toxics14060524)
Supplement: Supplementary file 1 [file toxics-14-00524-s001.zip › toxics-4333429-supplementary.pdf]

Table S1. Pollutant Classification and Corresponding LOD and LOQ Values

Category	LOD	LOQ
PFCA		
Perfluorobutanoic acid (PFBA)	0.02	0.06
Perfluoropentanoic acid (PFPeA)	0.02	0.06
Perfluorohexanoic acid (PFHxA)	0.01	0.03
Perfluoroheptanoic acid (PFHpA)	0.01	0.03
Perfluorooctanoic acid (PFOA)	0.01	0.03
Perfluorononanoic acid (PFNA)	0.01	0.03
Perfluorodecanoic acid (PFDA)	0.01	0.03
Perfluoroundecanoic acid (PFUdA)	0.01	0.03
Perfluorododecanoic acid (PFDoA)	0.01	0.03
Perfluorotridecanoic acid (PFTrDA)	0.01	0.03
Perfluorotetradecanoic acid (PFTeDA)	0.01	0.03
Perfluorohexadecanoic acid (PFHxDA)	0.01	0.03
Perfluorooctadecanoic acid (PFOdA)	0.01	0.03
PFSA		
Perfluorobutanesulfonic acid (PFBS)	0.01	0.03
Perfluoropentanesulfonic acid (PFPeS)	0.01	0.03
Perfluorohexane sulfonic acid (PFHxS)	0.01	0.03
Perfluoroheptanesulfonic acid (PFHpS)	0.01	0.03
Perfluorooctanesulfonic acid (PFOS)	0.01	0.03
Perfluorononanesulfonic acid (PFNS)	0.01	0.03
Perfluorodecanesulfonic acid (PFDS)	0.01	0.03
Emerging PFAS		
4,8-dioxa-3H-perfluorononanoic acid (ADONA)	0.01	0.03
9-chlorohexadecafluoro-3-oxanonane-1-sulfonate (6:2 Cl-PFESA (F-53B))	0.01	0.03
9-chlorooctadecafluoro-3-oxanonane-1-sulfonate (8:2 Cl-PFESA)	0.01	0.03
Hexafluoropropylene oxide dimer acid (HFPO-DA (Gen-X))	0.01	0.03

Table S2. Levels of PFAS congeners in freshwater fish from Zhejiang province

PFAS congeners	N	Detection rate (%)	ng/g wet weight					
			Min	Mean	SD	P50	P95	MAX
PFBA	240	18.3	ND	1.41	4.18	0.03	11.4	32.4
PFOS	150	70.7	ND	1.00	2.47	0.33	2.78	20.8
PFUnDA	240	59.6	ND	0.78	2.19	0.20	3.09	28.7
PFDA	240	53.8	ND	0.44	0.86	0.15	2.07	7.72
PFTrDA	240	48.8	ND	0.33	1.27	0.15	1.31	18.6
6:2Cl-PFESA	124	50.0	ND	0.24	0.59	0.01	1.12	4.92
PFNA	240	43.3	ND	0.18	0.37	0.14	0.52	2.82
PFOA	240	35.4	ND	0.15	0.29	0.09	0.60	2.09
PFDoA	240	4.17	ND	0.15	0.46	0.05	0.48	4.73
PFHxA	240	7.08	ND	0.14	0.79	0.01	0.15	9.50
PFTeDA	240	11.7	ND	0.13	0.54	0.01	0.53	7.54
PFHxDA	240	7.08	ND	0.13	0.23	0.01	0.30	1.89
PFPeA	240	3.75	ND	0.12	0.14	0.01	0.30	0.30
HFPO-DA	18	83.3	ND	0.11	0.08	0.12	0.22	0.22
PFOdA	240	20.0	ND	0.10	0.42	0.01	0.15	5.51
ADONA	124	9.68	ND	0.07	0.21	0.01	0.59	1.07
PFHpA	240	5.00	ND	0.06	0.07	0.01	0.15	0.40
PFHxS	150	12.7	ND	0.03	0.11	0.01	0.17	0.85
8:2Cl-PFESA	124	24.2	ND	0.03	0.12	0.01	0.09	1.25
PFDS	150	2.67	ND	0.01	0.03	0.01	0.01	0.27
PFPeS	126	2.38	ND	0.01	0.02	0.01	0.01	0.26
PFHpS	126	3.97	ND	0.01	0.01	0.01	0.01	0.14
PFNS	126	3.17	ND	0.01	0.01	0.01	0.01	0.12
PFBS	150	3.33	ND	0.01	0.00	0.01	0.01	0.05
Total	240	77.5	ND	4.97	6.88	2.63	18.1	53.9

Table S3. The estimated EDI for PFAS congeners

PFAS congener	ng/kg bw per day						
	Mean	SD	P25	Median	P75	P95	Max
PFUnDA	0.0234	0.0490	0.0061	0.0120	0.0255	0.0751	2.33
PFOS	0.0211	0.0437	0.0008	0.0090	0.0244	0.0791	1.40
PFDA	0.0146	0.0217	0.0046	0.0088	0.0168	0.0451	0.593
PFNA	0.0126	0.0192	0.0040	0.0077	0.0146	0.0375	0.593
PFTrDA	0.0112	0.0182	0.0033	0.0066	0.0129	0.0346	0.593
PFOA	0.0077	0.0138	0.0021	0.0043	0.0089	0.0242	0.593
PFDoA	0.0077	0.0158	0.0010	0.0030	0.0087	0.0284	0.593
PFBA	0.0097	0.0247	0.0008	0.0026	0.0107	0.0380	1.19
PFPeA	0.0054	0.0230	0.0003	0.0007	0.0019	0.0271	1.19
PFHxDA	0.0030	0.0118	0.0002	0.0005	0.0014	0.0144	0.593

PFHxA	0.0031	0.0118	0.0002	0.0004	0.0011	0.0152	0.593
PFOdA	0.0031	0.0119	0.0002	0.0004	0.0011	0.0153	0.593
PFTeDA	0.0027	0.0115	0.0002	0.0004	0.0010	0.0136	0.593
PFHpA	0.0027	0.0115	0.0002	0.0004	0.0010	0.0135	0.593
PFBS	0.0005	0.0007	0.0001	0.0003	0.0005	0.0014	0.0198
PFPeS	0.0005	0.0007	0.0001	0.0003	0.0005	0.0014	0.0198
PFHxS	0.0005	0.0007	0.0001	0.0003	0.0005	0.0014	0.0198
PFHpS	0.0005	0.0007	0.0001	0.0003	0.0005	0.0014	0.0198
PFNS	0.0005	0.0007	0.0001	0.0003	0.0005	0.0014	0.0198
PFDS	0.0005	0.0007	0.0001	0.0003	0.0005	0.0014	0.0198
HFPO-DA	0.0084	0.0134	0.0024	0.0050	0.0097	0.0258	0.464
6:2Cl-PFESA	0.0047	0.0131	0.0003	0.0011	0.0037	0.0199	0.279
8:2Cl-PFESA	0.0005	0.0008	0.0002	0.0003	0.0006	0.0017	0.0198
ADONA	0.0005	0.0007	0.0001	0.0003	0.0005	0.0014	0.0198

Table S4. The estimated EDI for Σ_{24}PFAS dietary exposure.

Age group	N	ng/kg bw per day					
		Mean	SD	P50	P90	P95	MAX
3-6	251	0.21	0.37	0.12	0.41	0.73	4.87
7-13	410	0.19	0.32	0.10	0.36	0.62	4.60
14-17	258	0.14	0.17	0.08	0.30	0.47	1.26
18-59	4762	0.14	0.27	0.08	0.30	0.46	12.19
≥60	1082	0.13	0.24	0.07	0.26	0.39	4.69
total	6763	0.14	0.27	0.08	0.31	0.47	12.19

Table S5. The estimated EDI for Σ_4PFAS dietary exposure.

Age group	N	ng/kg bw per day					
		Mean	SD	P50	P90	P95	MAX
3-6	251	0.06	0.09	0.04	0.16	0.20	0.84
7-13	410	0.05	0.08	0.03	0.13	0.17	0.79
14-17	258	0.04	0.05	0.02	0.09	0.13	0.39
18-59	4762	0.04	0.06	0.02	0.09	0.13	2.09
≥60	1082	0.04	0.09	0.02	0.07	0.12	1.87
total	6763	0.04	0.07	0.02	0.09	0.14	2.09

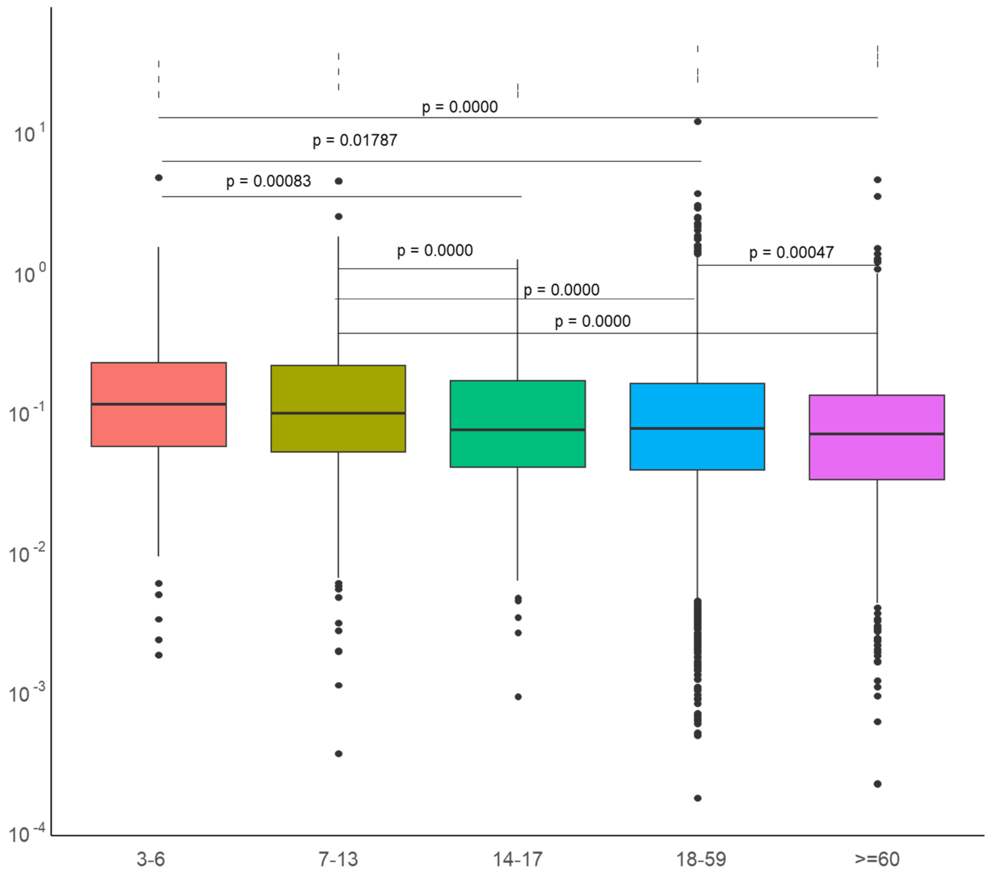

Figure S1. Comparison of ∑24PFAS exposure levels(ng/kg bw per day) among different age groups.

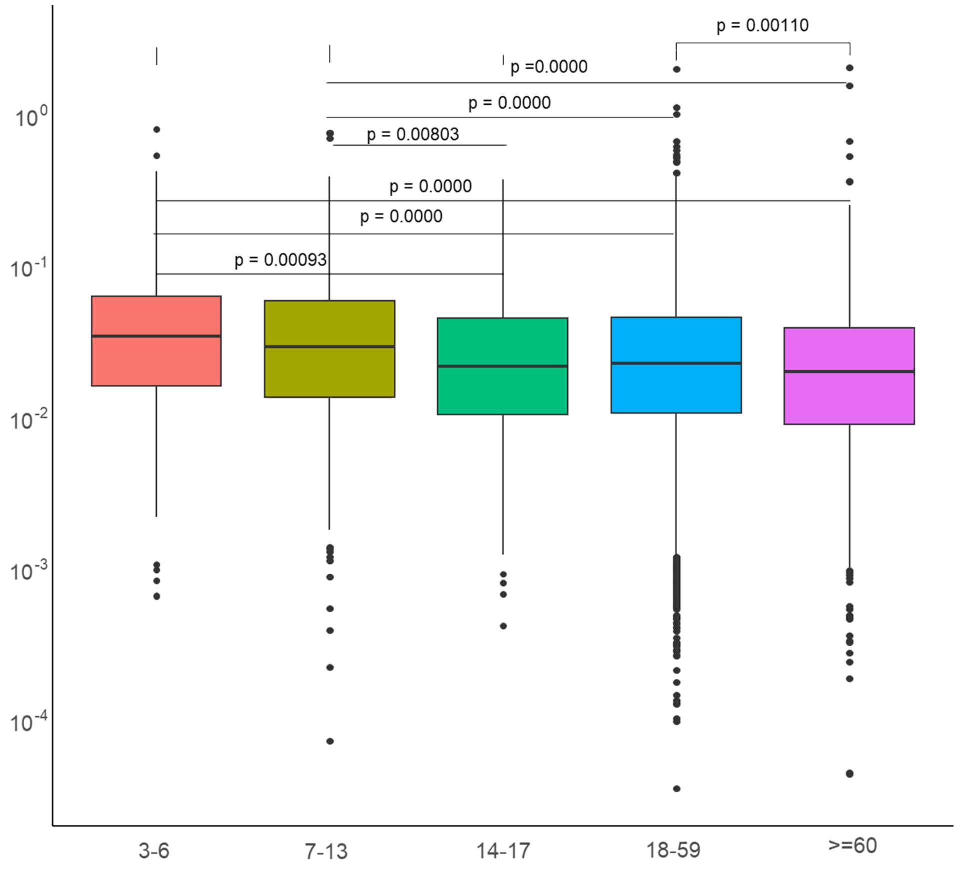

Figure S2. Comparison of $\sum_4$PFAS exposure levels(ng/kg bw per day) among different age groups.
